# Treating verbal working memory in a boy with intellectual disability

**DOI:** 10.3389/fpsyg.2015.01091

**Published:** 2015-07-30

**Authors:** Margherita Orsolini, Sergio Melogno, Nausica Latini, Roberta Penge, Sara Conforti

**Affiliations:** ^1^Dipartimento di Psicologia dei Processi di Sviluppo e Socializzazione, Sapienza Università di RomaRoma, Italy; ^2^Dipartimento di Pediatria e Neuropsichiatria Infantile, Sapienza Università di RomaRoma, Italy; ^3^Dipartimento di Psicologia, Sapienza Università di RomaRoma, Italy

**Keywords:** intellectual disability, training, attention, inhibition, switching, verbal working memory

## Abstract

The present case study investigates the effects of a cognitive training of verbal working memory that was proposed for Davide, a 14-year-old boy diagnosed with mild intellectual disability. The program stimulated attention, inhibition, switching, and the ability to engage either in verbal dual tasks or in producing inferences after the content of a short passage had been encoded in episodic memory. Key elements in our program included (1) core training of target cognitive mechanisms; (2) guided practice emphasizing concrete strategies to engage in exercises; and (3) a variable amount of adult support. The study explored whether such a complex program produced “near transfer” effects on an untrained dual task assessing verbal working memory and whether effects on this and other target cognitive mechanisms (i.e., attention, inhibition, and switching) were long-lasting and produced “far transfer” effects on cognitive flexibility. The effects of the intervention program were investigated with a research design consisting of four subsequent phases lasting 8 or 10 weeks, each preceded and followed by testing. There was a control condition (phase 1) in which the boy received, at home, a stimulation focused on the visuospatial domain. Subsequently, there were three experimental training phases, in which stimulation in the verbal domain was first focused on attention and inhibition (phase 2a), then on switching and simple working memory tasks (phase 2b), then on complex working memory tasks (phase 3). A battery of neuropsychological tests was administered before and after each training phase and 7 months after the conclusion of the intervention. The main finding was that Davide changed from being incapable of addressing the dual task request of the listening span test in the initial assessment to performing close to the normal limits of a 13-year-old boy in the follow-up assessment with this test, when he was 15 years old.

## Introduction

According to an influential multi-component model (Baddeley and Hitch, 1974; Baddeley, 2000, 2010), working memory consists of a central executive whose limited capacity attentional control is responsible for the active maintenance and processing of task-relevant information, which is temporarily held in domain-specific verbal and visuospatial stores or a multi-modal episodic buffer (Baddeley, 2000). Consistent with this model is the description of the central executive as a cluster of executive functions whose specific control process consists in updating the contents of working memory, switching between different tasks or procedures, inhibit irrelevant information or actions, coordinating multiple tasks (Baddeley, 1996; Miyake and Friedman, 2012).

The critical role that working memory plays in enhancing cognitive development is suggested by several studies that point to a strong relationship between executive functions, working memory, and fluid intelligence (see the recent review by Titz and Karbach, 2014) both in adults (Friedman et al., 2006; Wang et al., 2013) and children (Engel de Abreu et al., 2010; Giofre et al., 2013).

Learning is also promoted by working memory capacities and executive functioning, as suggested by a number of studies showing high correlations between working memory and measures of learning and academic achievement (Alloway and Passolunghi, 2011; Swanson and Alloway, 2012; Alloway et al., 2013). Working memory capacity is an effective predictor of performance in reading (de Jong, 1998; Gathercole and Pickering, 2000; Swanson, 2003; Gathercole et al., 2006) and mathematics (Gathercole and Pickering, 2000; Bull and Sherif, 2001; Geary et al., 2004). The executive processes of updating and shifting are also associated with scholastic attainment scores and performance on tests of reading and mathematics (St Clair-Thompson and Gathercole, 2006; Yeniad et al., 2013). Working memory and executive functions not only play a key role in learning but also affect a range of everyday life situations (e.g., following instructions or carrying out a sequence of actions) in which cognitive processing has to be complemented by short-term storage (see Gathercole and Alloway, 2006).

The strong association between working memory and executive functions on one hand and academic learning on the other hand is also shown by children with intellectual disabilities (ID) and borderline intellectual functioning (Numminen et al., 2000; Henry and Winfield, 2010; Poloczek et al., 2012).

Given the strong relationship that working memory and fluid intelligence have in typically developing individuals, we may ask whether working memory, especially when central-executive-loaded tasks are employed, is an area of weakness in the neuropsychological profile of children with ID. Most studies assessing working memory in children with ID analyzed their performance using age-expected norms and found deficits in all the subcomponents of working memory (Henry, 2001; Pickering and Gathercole, 2004; Van der Molen et al., 2007; Maehler and Schuchardt, 2009). Some studies have asked whether children with ID show lower performance compared to children of the same chronological age only (CA controls) or also to younger children with the same mental age (MA controls). This double comparison is assumed to distinguish the effects of a simple developmental delay from the effects of specific structural impairments in one of the components of the working memory system. Using this method, Van der Molen et al. (2009) assessed visual and verbal working memory in a group of children with mild intellectual disabilities (IQ 55–85) and found an unbalanced profile between the visuospatial and verbal components of the working memory system. Specifically, visuospatial working memory (tested with the *odd-one-out task*) was delayed compared to CA controls only, whereas performance in a verbal dual task involving central executive resources (i.e., listening span test), was lower compared to both the CA and MA controls. Other studies, however, found a reverse pattern in which non-verbal WM was delayed compared to the MA controls whereas verbal WM, always assessed with the listening span test, was lower only when compared to the CA controls (Danielsson et al., 2012).

As far as the verbal component of working memory is concerned, there is rich evidence that the phonological loop component of WM is weaker compared to mental age peers in most children with ID (Jarrold et al., 2000; Henry and MacLean, 2002; Van der Molen et al., 2009; Schuchardt et al., 2010, 2011) and even in children with borderline intellectual functioning (Henry, 2001; Henry and MacLean, 2002; Hasselhorn and Maehler, 2007).

Studies of executive functions in children with ID or borderline intellectual functioning are consistent in showing lower performance than chronological age comparisons (Conners et al., 1998; Levén et al., 2008; Alloway, 2010). A study assessing executive functioning with a comprehensive battery of tests (Danielsson et al., 2012) found that children with ID had lower performance than chronological age controls on all the executive function tests. Moreover, on the inhibition and planning tasks children with ID performed more poorly than the mental age comparison group. An inhibition deficit, mostly consisting in behavioral inhibition and interference control, emerged in a recent meta-analytic study (Bexkens et al., 2014) and generalized inhibitory difficulties were observed in a recent study on children with Down Syndrome (Borella et al., 2013).

In summary, children with ID or borderline intellectual functioning show heterogeneous domain-specific effects in performance with working memory tasks (Van der Molen et al., 2007). Such effects are likely to be related to disorder-specific “structural” impairments affecting a short-term storage of verbal, visual, or spatial information (Jarrold et al., 1999, 2006; Lanfranchi et al., 2004). Although working memory tasks may be more easily performed in one or the other domain, it is still an open question whether devoting attentional resources to processing current information, while simultaneously storing target items in memory to be retrieved later can be successfully treated in children with intellectual disabilities (see Perrig et al., 2009).

We review the evidence concerning such issue along with a discussion of the factors that generate relevant differences in WM training methods and their effects. Starting with the distinction between *strategy training* and *core* (Morrison and Chein, 2011) or process-based (Jolles and Crone, 2012) *training*, some programs teach strategies to facilitate the encoding and recall of more information, whereas other training approaches aim to induce changes in the target ability through extensive and repeated practice. Strategy training has been used to teach rehearsal in order to improve short-term memory (e.g., increases in digit span) in children with Down syndrome (Broadley and MacDonald, 1993; Comblain, 1994) or fetal alcohol spectrum disorder (Loomes et al., 2008). Rehearsal can prevent the quick decay of representations from the phonological loop in working memory and compensate for structural impairments of short-term storage. It is clear that children with intellectual disabilities can learn rehearsal and improve their memory span when they use such a strategy. However, the near transfer effects of this method—that is, the improvement that can be generated in similar but untrained tasks—have been little investigated; thus it is unclear, for instance, whether rehearsal can be used spontaneously in tasks that are similar but not identical to the trained task (e.g., from rehearsing digits to rehearsing words). Also unclear is whether rehearsal strategies can produce improvements in the parallel tasks of processing, memory encoding, and recalling that are involved in working memory.

Unlike strategy training, *core training* addresses the functionality of a mechanism through practice and repetition, as when cumulative rehearsal is intensively practiced to enlarge the storage capacity of the phonological buffer. When training Down syndrome children with overt cumulative rehearsal (e.g., if I said “car,” and you said “ball,” I have to say “car, ball”) for one or two 3−month periods, Conners et al. (2008) found effects on the digit span task and an increased phonological similarity effect, suggesting a deeper phonological encoding of information in short-term memory. However, when the task required subjects to both process and store information, no transfer was observed.

This finding led us to another crucial question regarding the characteristics of training methods—whether they address the central executive or only the short-term storage components of the working memory system. Some studies used computerized adaptive training to involve participants in processing current spatial (Jaeggi et al., 2011) or visual-auditory stimuli (Redick et al., 2013) and to decide whether they are the same (and/or have identical locations) as the n-back ones. Focusing on the near transfer effects of adaptive n-back training to tasks deeply involving the central executive (e.g., reading span), Redick et al. (2013) found that such transfer did not occur, whereas training complex span (Chein and Morrison, 2010; Harrison et al., 2013) produced near transfer to other central-executive-loaded working memory tasks. Complex span tasks, for instance, ask participants to recall a sequence of digits or pictures when there is a background processing task, such as counting or analyzing the orientation of the presented pictures. Such complex tasks involve crucial characteristics of the working memory system: allocating attentional resources to maintaining the task goals, storing relevant information, processing the current stimuli, and recalling target information in a sequentially ordered fashion.

A dual task involving processing the current stimuli (i.e., identifying which figure is the odd one), and remembering a target location across increasingly longer spans has been used by Van der Molen et al. (2010), using computerized training. A large group of adolescents with mild-to-borderline intellectual disabilities participated in either an adaptive or a stable training regimen with the visual dual task; a control group was trained with a single task. Results showed that children trained with dual tasks (no matter whether adaptive or stable) improved their performance in verbal short-term memory between pre- and post-testing. Visual working memory significantly improved only at follow-up testing, whereas performance with verbal working memory was not affected by training in any testing phase.

Soderqvist et al. (2012) analyzed the effects of a training procedure combining working memory and non-verbal reasoning (NVR) tasks. A sample of 41 children with ID participated in two training groups that used the same NVR tasks but differed regarding their treatment with either adaptive or non-adaptive, computerized, visual, simple-span tasks. There was large individual variability in the children's responses to intervention, and only children who made remarkable progress in the training tasks showed improved performance in verbal or visual working memory at post-testing. However, as there was no control group, it is not clear whether post-testing WM improvements in the subgroup of children who showed progress in the training tasks were an outcome of training and/or an outcome of repeated testing. Despite such methodological weakness, the findings of the study show that training success is feasible in children with ID and depends on the individual's modifiability in response to the increasing difficulties of the training regimen.

Bennett et al. (2013) used a computerized WM training consisting of visuospatial simple and complex span tasks. Children with Down syndrome aged seven to 12 years were allocated to either the intervention program or a waiting list group. Children in the intervention group significantly improved for visuospatial WM both immediately after the training and at 4-month follow-up but the training showed no effects on verbal WM.

In summary, there is evidence that using dual tasks in the visual domain can successfully improve visual working memory. Some studies have even found that such progress produced near transfer effects to verbal short-term memory in children with ID (Van der Molen et al., 2010). However, evidence that verbal working memory can be improved in children with ID, enabling them to effectively engage in verbal dual tasks, is still scarce.

In this single-case study we explore whether verbal working memory, assessed through a dual task such as the listening span test, can be improved as an effect of training in a child with a mild intellectual disability.

As the study's main goal is applicative, we designed a cognitive intervention that could be effective in practice and took into account the severe attention, impulsivity, and working memory difficulties of Davide, a 14-year-old boy with a diagnosis of mild intellectual disability. Our study explores whether a complex intervention can produce near transfer to an untrained task assessing verbal working memory (i.e., the listening span test) and whether effects on this and other target cognitive mechanisms (i.e., attention, inhibition, and switching) are long-lasting and can produce “far transfer” effects to cognitive flexibility.

## Background

Davide (a fictional name) was born in a middle-class family and started to show signs of motor delay before 1 year of age. The first formal assessment took place in a public neuropsychiatric unit when he was 3 years old, when he communicated mainly with gestures and showed a severe motor delay. As the child was very shy around peers and did not look people in the eye, the diagnosis at that time was global developmental disorder. After 2 years of treatment within a small group of children, his communication skills increased remarkably, and the diagnostic label was changed to that of a specific language impairment (evidenced in receptive language, verbal dyspraxia, and phonological disorder) associated with difficulties in emotion regulation and cognitive delay. Davide then attended speech therapy and entered primary school 1 year later than expected, assisted by a special educator who, according to Italian law, helps the children with special needs for a varying amount of time (according to the severity of their impairment) in regular classes. As the genetic analyses, the EEG and the functional magnetic resonance carried out by the family, never revealed any type of anomaly, Davide's parents have been swinging between believing that the child's cognitive weaknesses were generated by a learning disability that could be overcome in the future or considering the child's cognitive delay as a fixed characteristic. Davide seemed to have interiorized this latter conception and interpreted the difference in achievement between him and his peers at school as generated by insurmountable problems. He tended to present himself as a person “with problems” and was very prone to claiming his lack of intelligence whenever he realized to be incorrect.

Davide had received a diagnosis of mild intellectual disability of a non-specific etiology in three public neuropsychiatric units in Rome, showing an IQ ranging between 60 and 70 in different testing across the elementary and junior school years. The diagnosis was based not only on the intelligence quotient (IQ) level that was assessed with the WISC-III (Wechsler, 1991) but also on the level of adaptive functioning. Davide's social life was extremely poor. He had no relationships with school peers and did not have friends; although, he participated in activities at a Boy Scout center. Davide's life skills were also quite low as he had difficulties using money, traveling via metro or bus, planning his homework, and helping with simple works at home (e.g., setting the table). Academic learning had been assessed several times, with arithmetic skills and text comprehension corresponding to the level of an 8-year-old child in the last assessment when he was 13-year-old.

In our university clinical center, Davide was assessed when he was 14 years old and attending the third year of junior school. Turning to the results of our neuropsychological testing shown in Table 1, it is clear that language was still a core impairment, with performances in productive lexicon and receptive grammar below those of much younger children.

**Table 1:** **Davide's assessment before intervention (age: 14 years and 2 months)**.

**Test**		Performance (standard scores or percentiles^*^)
*VMI*—Developmental Test of Visual-Motor Integration (Beery and Butkenica, 1997)		
Visual test		In norm
Motor and visual-motor tests		5th percentile
*Arrows*—*Nepsy II* (Korkman et al., 2007)		−2
*Boston naming test* (Kaplan et al., 1983; Italian norms in Riva et al., 2000) (comparison with 10-year-old children, that is the highest age level of the test norms)		−1.78
*Peabody picture vocabulary test* (Dunn and Dunn, 1981; Italian norms in Stella et al., 2000)		−1
*Test of grammatical comprehension for children* (Chilosi et al., 1995) (comparison with 8-year-old children, that is the highest age level of the test norms)		Below the 10th percentile
*BVS—Battery for assessment of visual and spatial memory* (Mammarella et al., 2008)		
Simultaneous matrices (the child is asked to memorize the position of red circles in a matrix and reproduce it figuring out the position immediately below)		The task was too difficult and was not completed
Paths on a matrix (the child is asked to memorize the starting position of a symbol in a matrix and follow instructions to reproduce the arrival point)		−2.9
*Battery for neuropsychological assessment in adolescence* (Gugliotta et al., 2009)		
Direct digit span		In norm
Backward digit span		−1.26 (raw score: 3)
*Word repetition (from Word list interference)—Nepsy II* (Korkman et al., 2007)		−1.3
*Listening span test* (Pazzaglia et al., 2000) (comparison with children aged 11–13, that is the highest age level of the test norms)		
Number of words correctly recalled in order		Raw score: 0
Number of errors in judging sentences plausibility		−2.47
Number of intrusion errors (recalled words that do not occupy the sentence ending position)		Raw score: 0
*Episodic memory—TOMAL* (Reynolds and Bigler, 1994)		
Recall of stories—Number of recalled content units		1st percentile (raw score: 14)
Selective memory of words (immediate)		16th percentile
*Attention* (Di Nuovo, 2000)		
Alertness (*Simple reaction time*)		In norm
Selective attention (*Speed and accuracy*)—Errors		−0.66
Selective attention (*Speed and accuracy*)—Reaction time		−3.5
*Bells* (Italian norms of Biancardi and Stoppa, 1997)		
Selective attention		−1.5
Sustained attention		−4.5
*Fluency—Nepsy II* (Korkman et al., 2007)		
Phonological fluency		−0.33
Semantic fluency		−0.66
*Stroop test*, (Di Nuovo, 2000)		
Difference between baseline and condition with interference—Errors		−0.34
Difference between baseline and condition with interference—Reaction time		−2.4
*Inhibition*—*Nepsy II* (Korkman et al., 2007)		
*Naming* condition	Errors	In norm
	Completion time	1
*Inhibition* condition	Errors	Below the 2nd percentile (raw score = 7)
	Completion time	−2.6
*Switching* condition	Errors	Below the 2nd (raw score = 46);
	Completion time	1.33
*Animal sorting Nepsy II* (Korkman et al., 2007) Total correct sorts		−2.6
*Theory of mind Nepsy II* (Korkman et al., 2007)		In norm

* *Comparison with chronological age norms unless specified otherwise in the table*.

Verbal short-term memory was low but within normal limits, whereas complex dual tasks in both the spatial (see performances with the BVS battery by Mammarella et al., 2008 in Table 1) and the verbal domain of working memory could not be addressed in this initial assessment. In the listening span test (Pazzaglia et al., 2000), when he was asked to carry out the dual task of providing judgments of sentence plausibility and memory encoding of the last word of each sentence, Davide could not remember one word and only gave judgments of sentence plausibility; however, he made several errors. Such difficulties with dual tasks both in the verbal and visuo-spatial domains were likely to be related, on one hand, to the very low language and visuo-spatial processing skills (see the performances on sentence comprehension and with the visuo-spatial test ≪ Arrows ≫ in Table 1). On the other hand, the difficulties with attention, inhibition, and switching contributed to an impaired performance with executive-loaded working memory tasks. Selective attention (see Table 1) was, in fact, exceedingly slow, and among the executive processes there was a particularly low performance with inhibition, whereas the switching task had been addressed by Davide in a dysfunctional quick way that resulted in a huge number of errors (see Table 1).

Episodic memory (see Table 1) showed a different pattern of performance according to whether items to be recalled later were single words that could strengthen their representation through repetition (as in the test ≪ Selective Memory for Words ≫, Reynolds and Bigler, 1994) or were narrative contents to be recalled immediately after one single listening (as in ≪ Recall of Stories ≫, Reynolds and Bigler, 1994).

Davide's low processing speed emerged both in tests engaging executive control (see, for instance, the inhibition completion time) and in everyday life actions involving visual-motor coordination (e.g., exceedingly slow typing with the computer's keyboard) or discourse processing (e.g., long pauses before answering complex questions in conversation or following instructions).

Despite a poor social life and an extremely scarce experience of communicating with peers, Davide had a good performance on a theory-of-mind task (see Table 1), and his good ability of taking into account feelings and thoughts of other people was also clear from the conversations shared with him in the initial assessment (Fatigante et al., 2015).

Following ethical approval, informed written consent from the parents was obtained for Davide to include him in our experimental treatment. Davide was also involved in decisions concerning his participation in the training activities. When we proposed a treatment (“Would you like to exercise your attention and memory in our lab?”), Davide initially kindly declined our proposal: “Thank you. Everybody wants to give me some help, but I'm very busy with my studies and Boy Scout activities.” We then suggested he could try to come only three times and then make a final decision. He eventually decided to accept our proposal because, he said, “You can perhaps change my life.” We then clarified that we could not “change his life” but only teach him skills and give him support in his own attempts to change.

## Discussion

### Cognitive training program

Previous studies that have trained WM in children with ID used computerized tasks with structurally similar exercises that varied in terms of difficulty levels (Van der Molen et al., 2010; Soderqvist et al., 2012; Bennett et al., 2013). We assumed instead that cognitive enhancement may benefit more from training with varying tasks (Jolles and Crone, 2012) and that both progression from simple to complex tasks and change of stimuli could be important to boost the participants' motivation. Other key elements in our program included (1) core training of target cognitive mechanisms through repeated practice; (2) guided practice emphasizing concrete strategies to engage in exercises (e.g., verbalization to promote the task's goal maintenance); and (3) variable amount of the adult's support to adapt the task difficulty to the child's actual level of performance.

Core training through repeated practice was thus complemented in our training by adults leading verbal interaction and promoting an attentional control on the task's goal maintenance and the strategies that may help task execution. For instance, the adult asked the child to rephrase instructions, select characteristics on which to focus attention, anticipate possible sources of confusion in the task, and rehearse or visualize contents for later recall.

As illustrated in Table 2, our experimental training (occurring after the phase 1 control treatment) started from attention, as attention is involved in working memory (Vandierendonck, 2014), and it is known that weak attention skills are often present in children with ID, with a strong negative impact on working memory (Kirk et al., 2015). There were then activities related to inhibition that asked participants to process negative sentences to accomplish selection of target items (e.g., “The thief does not have blond hair”) or semantic categorization of pictures (e.g., “You cannot play cards with animals”). Processing of sentences with negation has been shown to involve the left inferior frontal gyrus (Bahlmann et al., 2011) that is also involved in tasks related to inhibition of irrelevant stimuli (Swick et al., 2008).

**Table 2:** **Phases of the cognitive training program**.

	**First 10-week unit—Phase 2a**		**Second 10-week unit—Phase 2b**	**First and second 8-week units—Phase 3**
	**Attention**	**Inhibition**	**Switching and simple verbal working memory**	**Complex working memory**
Adult's led interaction is focused on enhancing	Verbalization of stimuli Systematic visual exploration Sustained attention Selective attention	Maintenance of the task's goal Divided attention Selection of members of target categories	Rehearsal strategies Task planning and sequencing Focus on relevant information Semantic integration in sentence processing Summarizing the available information Anticipation of possible sources of difficulty Generalization of approach to different tasks	
Examples of computer-presented exercises and card games	• *Animal detective*: An incomplete picture appears on the computer screen and quickly disappears. The participant is asked to recognize the animal and then identify the lacking part of the picture, selecting it from four cards. • *Monsters*: An adult and child take turns in selecting one or more cards with monsters, describing their characteristics and communicating the precise location in which they put them. If the second player (who cannot see what the first is doing) makes the same choices as his/her companion does, the first player wins some points.	• *Characters detective*: A thief has been seen from people who describe his/her characteristics. Relying on each of such descriptions (e.g., “the thief was not a woman” or, “the thief did not wear glasses”), the participant removes images from a pool of suspects until the thief is identified. • *Category*: Each player has six cards and proceeds on a game of the goose board if he/she can play cards according to the category specified on the board box. Categories may be single or multiple (e.g., “food and furniture”) and affirmative or negative (e.g., “no fruits, no clothes”).	• *Guessing what*: The participant is asked to discover what the object hidden on the computer screen is by relying on the information provided by two types of characters. A wizard will say something that is opposite of the real characteristic (e.g., “if the wizard says that the thing is put on a lower part of the body, you have to think that it is put on an upper part of the body”). A pessimistic man will say something true but will add pessimistic evaluations that may distract you (e.g., “he will say that you wear this thing when it is hot, and he will add that if you do not do so, it may be very dangerous, and you can even die”). • *The dolphin game*: Players proceed with a game of the goose if they can repeat the sequence of words that has being said by the other player and add a new word according to the instruction specified on the board box. Boxes on the board ask for a fixed number of words (from 2 to 6) either starting with a given letter or belonging to a given category.	• *Stories*: Short narrative sequences are read by the adult and are also shown on the computer screen with the written text accompanied by a picture. For instance: “A hare was very proud of herself because she could run quickly. One day she said to all the other animals: - nobody is quicker than me; nobody has the courage to race with me-.” After the last sentence is read, the short passage disappears from the computer screen, and the participant is asked to produce a pragmatic judgment (e.g., “is what the hare says friendly?”) and then to recall the sentence. • *Take cards and remember*: Each player has three picture cards and can take one of four picture cards on the table, following the given rules (e.g., humans can take animals, animals can take plants or fruits, plants or fruits can take objects). At the end of the round, each player attempts to recall the word that was written on each of the taken cards (e.g., the word “surprise” written under the image of a birthday cake), and if he/she manages to do so, he/she wins the cards.

After the first 10-week unit, treatment was focused on both switching and simple working-memory tasks, with the former asking participants to practice different actions in the same exercise (e.g., looking at the picture and either saying something that was not true for that picture, or saying something that was true but different from the word that was written on the top of the picture). Phase 2b treatment also involved simple verbal working-memory tasks, asking participants to recall sequences of items belonging to a target semantic category or sequences of words starting with a target phoneme.

Phase 3 treatment engaged working memory with complex tasks that consisted either of verbal dual tasks where the participant is asked to recall information after having accomplished a different task (e.g., recalling a sentence after having judged whether that sentence was friendly or not) or tasks engaging inferential processes (e.g., guessing the place in which a short dialogue has occurred) after the content of a short passage had been encoded in episodic memory.

Each phase of the experimental training in our university lab consisted of 2-h weekly sessions that started with conversation and narrative talk to promote a close adult-child relationship and mitigate the shame feelings and self-undervaluation beliefs that are often present in children with ID. After such a warming stage, there was an exercise presented through PowerPoint and a card game that stimulated the target cognitive mechanisms of each phase (see Table 2 for examples of exercises and games).

### The research design

**Figure 1:**
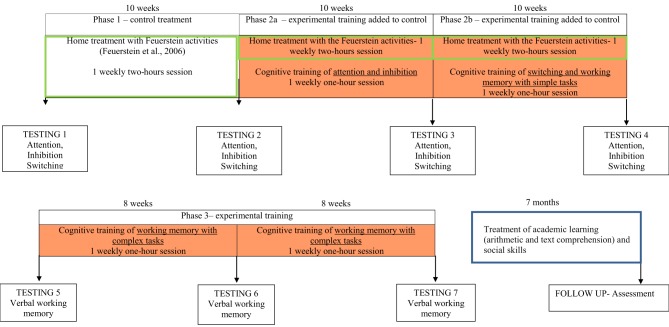
**The research design**.

As our program combines core training (i.e., repeated practice involving target cognitive mechanisms) and strategy training, we started with a control condition that was only focused on strategy training and involved the visuo-spatial domain. As illustrated by Figure 1, in phase 1 Davide received home training based on the Feuerstein approach (Feuerstein et al., 2006) and centered on visuo-spatial activities (e.g., “Organization of dots”). Learning how to inhibit impulsiveness, develop visual strategies, maintain visual attention to details, work to reach precision, and analyze sources of facilitation in task execution were the main objectives pursued through the Feuerstein approach. As each activity of phase 1 stimulated both selective and sustained attention but there was no repeated practice related to inhibition of response or switching, we predicted an effect on attention but no effect on inhibition and switching after phase 1.

The home treatment started to be accompanied by our experimental cognitive training program in the university lab that first stimulated attention and inhibition (phase 2a), then switching and working memory with simple tasks (phase 2b). Thus, in phases 2a and 2b, there was a combination of two treatments, the first being the continuation of the home treatment and the second being the specific stimulation of inhibition and switching in the verbal domain. If training effects were affected by the specific core training introduced in phases 2a and 2b, we should observe a different trend of improvements between these two phases, with performance in inhibition and switching higher after phases 2a and 2b respectively.

In phase 3, Davide was only involved in our experimental cognitive training of working memory with complex tasks for two subsequent 8-week time units. Davide's performance with the listening span test was assessed at the beginning of this phase and then at the end of each treatment unit. Phase 3 allowed us to compare the effects of a specific stimulation of working memory with complex tasks (testings 6 and 7) with those generated by the preceding phases (testing 5). Eventually, after a 7-month delay in which Davide was involved in a treatment of academic learning (namely, arithmetic, and text comprehension) and social skills, a follow-up assessment was carried out to explore whether the effects observed immediately after our training units were long-lasting, independent from the experience of being repeatedly tested with the same tasks, and generalizable to a task-assessing cognitive flexibility that was completely different from the type of tasks used in the training.

### Assessing the immediate effects of the cognitive training program

Davide's assessment was carried out with Italian tests that either have been adapted from international tests (e.g., Nepsy II, Korkman et al., 2007) or have been designed in Italian (e.g., *Attention* by Di Nuovo, 2000). Each test used in our study has been validated with Italian participants and has good reliability.

Whereas Davide's initial assessment has been quite comprehensive, including different domains and abilities, the evaluation of the effects of our cognitive training program focused on attention, inhibition, switching, and verbal working memory.

#### Inhibition and switching (testings 1–4 and follow-up)

In this timed test of the Nepsy II battery (Korkman et al., 2007), the ability to inhibit automatic responses in favor of novel responses and the ability to switch between response types is assessed. In the Naming phase of the task, the participant looks at a series of black and white shapes (circle and square) or arrows (pointing up and down) and names either the shape or the direction. In the Inhibition phase, the child names the same symbols but is asked to apply the non-target label (e.g., saying “square” for a circle or “up” for an arrow pointing down). In the Switching phase, the child is asked to say the correct name for black symbols but to apply the non-target label if the symbol is white (e.g., “down” for a white arrow pointing up or “circle” for a white square). The completion time and the total number of mistakes (including self-corrections) are evaluated for naming, inhibition, and switching.

#### Verbal working memory (testings 5–7 and follow-up)

Verbal WM was assessed with the Listening span test, an Italian adaptation (Pazzaglia et al., 2000) of the Daneman and Carpenter (1980) task consisting of sentences that are auditorily presented in blocks of increasing span (from two to six). The participant is asked (i) to judge the plausibility of each sentence (state whether it is true or false) and (ii) to recall the last word of each sentence, in the correct order, at the end of each block. The total number of words correctly recalled *in order* provides one type of score. For instance, if a subject is presented with a six-span block and recalls the last word of the third and fourth sentences in the right order, the score in this block would be 2. Further types of score are the number of errors with sentence judgements and the number of intrusion errors (recalling words that are not the last in the sentence).

### Assessing the long-term effects of the cognitive training program (follow-up testing)

#### Attention

Selective attention was evaluated using a task from a computerized battery (Di Nuovo, 2000). Participants are shown a sequence of numbers on the computer screen, and as soon as one of the numbers becomes surrounded by a red circle, they have to press the corresponding number on the computer keyboard; the reaction time and errors are evaluated.

#### Inhibition and switching

Inhibition was evaluated with a test assessing interference control (Di Nuovo, 2000) through an adjustment of the classic Stroop test. The computerized test consists of two sequential tasks. The first is baseline condition—asking the participant to name colored squares—and the second is interference condition, asking the participant to name the ink color of the printed color words. The difference between the scores obtained in the first condition and second condition measures the subject's ability to overcome the distraction induced by irrelevant stimuli. Inhibition and Switching were also assessed with the Nepsy II test (see the description in the previous section).

#### Short-term memory, working memory, and episodic memory in the language domain

A Forward digit span (Gugliotta et al., 2009), in which the examiner reads a list of numbers—a digit per second—and the participant must immediately repeat them back, was used to evaluate verbal short-term memory. The starting point in the task is a three-digit list, and the span is increased until the participant fails in all three lists of the same span. The score is the highest span in which the child manages to correctly repeat two out of three lists of that span. Verbal short-term memory was also tested with a word span using the first part of the test Word Interference from the Nepsy II. The child is auditorily presented with blocks of words increasing in span (from two to five) and is asked to repeat them in the same order. The number of blocks correctly repeated is the task score. Verbal working memory was assessed both with the Listening span test (see the description in the previous section) and a simple task, Backward digit span (Gugliotta et al., 2009), which is similar to a Forward digit span in the presentation of the items and score assignment, but at the end of each sequence, the child is asked to recall the presented digits in the reverse order.

Episodic memory was evaluated with Memory for Stories, a subtest of the Test of Memory and Learning (Reynolds and Bigler, 1994). Participants are asked to recall three short-story passages that were read by the examiner. Credit is given for each element of the story repeated correctly, irrespective of whether recall is verbatim or in a sequence that is different from the heard story. Only immediate memory was assessed.

#### Cognitive flexibility

The test Animal Sorting from the Nepsy II (Korkman et al., 2007) was used to assess concept formation and the ability to shift from one concept to another. The child sorts pictures cards as quickly as possible into two groups of four cards each, using self-initiated criteria.

### Results

Table 3A shows the results on selective and sustained attention assessed through the Bells test (Biancardi and Stoppa, 1997). Davide made a noticeable progress (almost one standard deviation on selective and about three standard deviations on sustained attention) after the home treatment with the Feuerstein activities (testing 2). As such activities promoted a systematic exploration of visual stimuli and a top-down search for characteristics (e.g., four equidistant dots) that can identify target shapes (e.g., a square), it is understandable that such activities enhanced attention. Davide's performance with selective and sustained attention continued to improve from testings 2–4 (see sustained attention improving of about one standard deviation in testing 3).

**Table 3A:** **Effects of treatment on attention, inhibition, and switching**.

	**Testing 1**	Testing 2^*^	Testing 3^**^	Testing 4^***^
**Selective and sustained attention (*Bells*, Italian norms in Biancardi and Stoppa, 1997) analyzed with standard scores (chronological age norms)**				
Number of targets identified in the first 30 s	−1.5	−0.46	−0.52	0.46
Number of targets identified in 240 s	−4.5	−1.59	−0.15	−0.31
**Inhibition (Korkman et al., 2007) analyzed with percentile ranks and standard scores (chronological age norms)**				
Errors—Percentile ranks	< 2 (raw score: 7)	< 2 (raw score: 8)	>75 (raw score: 0)	>75 (raw score: 0)
Completion time—Standard scores	−2.6 (raw score: 106)	−2 (raw score: 81)	−1.33 (raw score: 70)	−1.33 (raw score: 69)
**Switching (Korkman et al., 2007) analyzed with percentile ranks and standard scores (chronological age norms)**				
Errors—Percentile ranks	< 2 (raw score: 46)	< 2 (raw score: 26)	< 2 (raw score: 13)	Between the 11th and the 25th percentile rank (raw score: 9)
Completion time—Standardized scores	1.33 (raw score: 59)	−2 (raw score: 118)	−2.6 (raw score: 175)	−2.6 (raw score: 157)

* After 10 weeks of home treatment with Feuerstein activities;

** after 10 weeks of the cognitive training program added to the home treatment;

*** *after further 10 weeks of the cognitive training added to the home treatment*.

Turning to Davide's performance with inhibition, Table 3A shows that there was a remarkable change after the first 10 weeks of our cognitive training program (testing 3). Davide changed from being under the second percentile rank for correctness in testing 2, to being in norm in testing 3, whereas his completion time was still high but within normal limits in the same phase. Thus, only when a specific stimulation of inhibition was added to the Feuerstein treatment did Davide improve on a test assessing this type of executive function.

Results on switching are again suggestive of an effect of specific stimulation. Focusing on the initial assessment, Davide not only failed to maintain the task rules but also underestimated the task difficulty as he tried to be very quick. After the home treatment with the Feuerstein activities (testing 2), he still made an extremely high number of errors but seemed to realize that the switching task was difficult and required slowness. Only after the second phase of our cognitive training program, when switching had been specifically stimulated (testing 4), did Davide's performance on switching improve for correctness, whereas the completion time was still much higher than chronological age norms.

Turning to the findings concerning verbal working memory in Table 3B, an improvement occurred after the 30 weeks of treatment. Davide's performance with the listening span test shifted from being only focused on providing judgments of sentences' plausibility in the initial assessment to accommodating the dual task request in testing 5. Despite such progress, Davide's performance was still extremely low in terms of number of words correctly recalled in order, and the intrusion errors were exceedingly numerous. However, after only 8 weeks of training that stimulated verbal working memory with complex tasks (see testing 6 in Table 3B), Davide's improvement increased by more than one standard deviation from the previous testing. In terms of raw scores, whereas Davide had changed from recalling 0 words to correctly recall 7 words after the first 30 weeks of treatment, he improved on 8 more words (from 7 to 15 words correctly recalled in order) after 8 weeks of specific training. After a further 8 weeks of training, the number of words correctly recalled slightly decreased (see testing 7), whereas performance with both intrusion errors and sentence judgments further improved in this last assessment.

**Table 3B:** **Effects of treatment on verbal working memory analyzed with standardized and raw scores (listening span test, Pazzaglia et al., 2000)**.

	**Testing 1**	Testing 5^*^	Testing 6^**^	Testing 7^***^
Number of words correctly recalled in order	^∧∧^	−3.12 (raw score: 7)	−1.57 (raw score: 15)	−2.28 (raw score: 12)
Number of errors in judging sentences plausibility	−2.47 (raw score: 8)	−0.89 (raw score: 4)	−1.29 (raw score: 5)	−0.10 (raw score: 2)
Number of intrusion errors	^∧∧^	−9.17 (raw score:13)	−6.90 (raw score:10)	−4.90 (raw score: 7)

* After 30 weeks of treatment focused both on Feuerstein activities and training of inhibition, switching and working memory with simple tasks;

** after 8 weeks of training with complex memory tasks;

*** *after further 8 weeks of training with complex memory tasks*.

∧∧ *The test asks to recall the last word of each sentence in blocks of increasing length (from 2 to 6 sentences) but Davide did not try to recall one word and for this reason he did not make intrusion errors either*.

Despite the noticeable improvements, difficulties in carrying out a dual task asking to semantically process sentences and to memory-encode some target information were still present. We should remember that Davide was 15 years old in testing 7, whereas the highest age level in the Italian listening span test is 11–13. More than fifty percent of the subjects in the test's normative sample made 0–1 intrusion errors (Pazzaglia et al., 2000). As such types of errors consist in recalling words that do not occupy the sentence ending position (e.g., recalling “football” instead of “mountain” for the sentence *Football is a sport that you can only practice in a high mountain*), it is clear that the high number of intrusion errors still produced by Davide in testing 7 was an indicator of difficulties in inhibiting irrelevant information.

Listed in Table 4 are the scores that are more than two standard deviations below mean, or at the fifth percentile, before and after the control treatment (testing 2), the training stimulating attention, inhibition and switching (testings 3–5), and the training stimulating WM with complex tasks (testings 6–7). We applied to this list of performances evaluated with standard scores or percentile ranks the line of reasoning that Parker et al. (2007) considered for raw scores when they defined the “percent of all non-overlapping data” (PAND) as the percent of all data remaining after removing the number of data points that overlap between a baseline and an intervention phase. Applying this same argument, we asked how many “deficit” scores on tests assessing the cognitive mechanisms that were the target of our training did not overlap before and after intervention. Only sustained attention improved above the criteria level after the control treatment (testing 2); there were three out of eight overlapping data after experimental training of attention, inhibition, switching, and working memory with simple tasks. The switching completion time, and two scores of the listening span test, remained in fact below the criterial level in testings 3–5. After experimental training of working memory with complex tasks (testing 6–7) the number of words correctly recalled in order in the listening span test improved above the criteria level in the first treatment unit (testing 6), whereas intrusion errors remained below the criterial level. Pooling together the number of scores improving after the different phases of experimental training, there were six out of eight non-overlapping data. Percentage of non-overlapping data (PAND) for our experimental training was therefore 75%.

**Table 4:** **List of Davide's performances before and after different phases of treatment**.

	**Initial assessment**	**Control treatment (Testing 2)**	**Experimental training added to the control treatment (testings 3–4, 5)**	**Experimental training only (testings 6–7)**
Scores that are 2 standard deviations below mean (or below the 5th percentile)	• Sustained attention • Inhibition errors • Inhibition completion time • Switching errors • Switching completion time • Number of words correctly recalled in sequence ^*^ • Errors in judging sentence plausibility^*^ • Intrusion errors^**^	• Inhibition errors • Inhibition completion time • Switching errors • Switching completion time	• Switching completion time • Number of words correctly recalled in sequence • Intrusion errors	• Intrusion errors
Scores that are within normal limits (less than 2 standard deviations below chronological age mean or above the 10th percentile)		• Sustained attention	• Inhibition errors • Inhibition completion time • Switching errors • Errors in judging sentence plausibility	• Number of words correctly recalled in sequence

* *These performances were evaluated in the initial assessment and then in testings 5–7*.

** *We infer that these errors would correspond to the deficit range in the initial assessment, as Davide was only able to judge sentence plausibility but did not recall any word in the listening span test*.

Turning to the results of the follow-up testing that was run when Davide was 15 years old, it can be observed in Table 5 that after 7 months in which there was no specific exercise of attention, inhibition, switching, and verbal working memory, a number of training effects were still observable even when they could be assessed through tasks that were different from the ones used throughout the treatment phases. Selective attention was tested with a computerized task and was in norm; interference control was also tested with a computerized Stroop test and was in norm for both errors and reaction times. Inhibition and switching were again tested with the Nepsy II tasks and were in norms in terms of correctness, but below norms for completion times.

**Table 5:** **The initial and follow-up assessments analyzed with standard scores or percentile ranks (comparison with chronological age norms unless specified otherwise in the table)**.

		**Initial assessment (age: 14 years and 2 months)**	**Follow-up assessment (age: 15 years and 10 months)**
**ATTENTION**			
*Selective attention* (Di Nuovo, 2000)			
Errors		−0.66	−0.66
Reaction times		−3.5	−1.42
**INHIBITION AND SWITCHING**			
**Interference control (Stroop Test, Di Nuovo, 2000)**			
Difference between baseline and condition with interference—Errors		−0.34	−0.34
Difference between baseline and condition with interference—Reaction time		−2.4	0
**Inhibition**—**Nepsy II (Korkman et al., 2007)**			
Errors		Below the 2nd percentile rank (raw score = 7)	Between the 51st—75th percentile rank (raw score: 1)
Completion time		−2.6	−2
**Switching—Nepsy II (Korkman et al., 2007)**			
Errors		Below the 2nd percentile (raw score: 46)	Above the 75th percentile rank
Completion time		1.33	−2.33
**SHORT-TERM MEMORY, WORKING MEMORY, AND EPISODIC MEMORY IN THE LANGUAGE DOMAIN**			
**Short-term memory**			
*Direct digit span* (Gugliotta et al., 2009)		−0.1 (raw score: 5)	−1.7 (raw score: 4)
*Word repetition* (from Word list interference)—*Nepsy II* (Korkman et al. 2007)		−1.3 (raw score: 14)	−0.66 (raw score: 16)
**Working memory**			
*Backward digit span* (Gugliotta et al., 2009)		−1.26 (raw score: 3)	−1.13 (raw score: 3)
*Listening span test* (Pazzaglia et al., 2000)^*^	Number of words correctly recalled in order	^∧∧^(raw score: 0)	−0.76 (raw score: 21)
	Number of errors in judging sentences plausibility	−2.47 (raw score: 8)	−0.10 (raw score: 2)
	Number of intrusion errors (recalled words that do not occupy the sentence ending position)	^∧∧^	−3.89 (raw score: 6)
**Episodic memory**			
*Recall of stories* (Reynolds and Bigler, 1994) Number of recalled content units		1st percentile (raw score: 14)	9th percentile (raw score: 29)
**COGNITIVE FLEXIBILITY**			
*Animal sorting—Nepsy II* (Korkman et al., 2007) Total Correct Sorts		−2.6 (raw score: 2)	−1.6 (raw score: 4)

∧∧ *The test asks to recall the last word of each sentence in blocks of increasing length (from 2 to 6 sentences) but Davide did not try to recall one word and for this reason he did not make intrusion errors either*.

* *Comparison with children aged 11–13, that is the highest age level of the test norms*.

Performance with the listening span test was within the norms of junior school children (age range: 11–13) in terms of a number of words that were correctly recalled in sequence and correct sentence judgments, whereas intrusion errors were still much above the mean of the same age range.

The long-term sustainment of improved performances in the listening span test were not accompanied either by an improvement of verbal short-term memory (see standard scores of direct digit span and word repetition in Table 5), nor by a better episodic memory.

The follow-up testing showed an improvement in Davide's cognitive flexibility. His performance in Animal Sorting test (Korkman et al., 2007) shifted from 2.6 to 1.6 standard deviations below the chronological age mean. This test asks participants to sort pictures into two groups of four using various self-initiated sorting criteria and engages both concept formation and shifting.

### Recapitulating the findings of different testing phases

In the current study, we assumed that attentional control and executive functions of inhibition and switching are all involved in verbal working memory, and for this reason we structured a complex treatment that stimulated such functions before engaging the ability to address verbal dual tasks. Davide's training started with a control condition based on the Feuerstein approach (Feuerstein et al., 2006) and centered on visuo-spatial activities. As learning how to inhibit impulsiveness, maintaining visual attention to details, and working to reach precision were pursued in these activities, Davide showed a remarkable increase in sustained visual attention after this control treatment phase, but did not show improvements in his severely impaired performances with the inhibition and switching tests. After our experimental cognitive training program focusing on attention and inhibition was added to the treatment with the Feuerstein activities, Davide's performance with sustained attention continued to improve in about one standard deviation—whereas performance with inhibition changed from being under the second percentile rank for correctness and two standard deviations below norms for completion time—to being in the norm for correctness and still low but within normal limits for completion time.

Again, only after our experimental cognitive training program stimulated switching in the subsequent phase did Davide's performance on switching changed from being severely incorrect to being within normal limits for correctness, whereas his completion time was still much below chronological age norms.

When verbal working memory was assessed again after this combined treatment phases, Davide's performance was still severely impaired; although, he managed to accommodate the dual task request. After the first 8-week unit of training with complex working memory tasks, Davide was evaluated again with the listening span test. The number of words that were correctly recalled in sequence was low but close to normal limits, the number of errors in judging the sentences' plausibility was within normal limits whereas intrusion errors were still very high. In the second 8-week unit of training, Davide's performance slightly decreased for the number of words correctly recalled but improved for the other two parameters.

Overall, there were six out of eight scores that shifted from being more than two standard deviations below the chronological age mean (or below the fifth percentile rank) in the initial assessment to being either within or close to normal limits after the specific stimulation of target cognitive mechanisms introduced in each phase of Davide's experimental treatment.

### Effects of training or repeated testing?

Although these findings are very encouraging, how can we rule out that increasing exposure to tests, rather than training, was the factor generating changes of target cognitive mechanisms?

First, the findings described in the previous section suggest that a predicted change in the dependent variable covaries with manipulation of the independent variable (Kratochwill et al., 2010). In other words, improvements of specific cognitive functions were observed only after a phase in which a specific stimulation of that function had been introduced. Second, most of the observed improvements were maintained in the follow-up assessment, after 7 months in which attention, inhibition, switching, and verbal working memory had not been tested anymore. Third, the same experimental cognitive training program in which Davide was involved produced similar effects in a multiple case study in which such training was implemented in a more intensive way and contrasted with a control training (Orsolini et al., 2014). In such a study, six children with ID or “borderline intellectual functioning” were tested before and after a 10-week treatment, consisting of either our experimental program or a control training focused on narrative skills. Each child in this study was tested twice, and we found that each of the three children involved in the experimental program improved by at least one standard deviation in the listening span test, whereas only one of the three children participating in the control group showed a similar improvement.

Thus, the findings of the current study suggest that a combined intervention, in which a core training of specific cognitive mechanisms interacted with teaching a strategic approach to task execution, was effective in improving Davide's cognitive performances. Although our research design did not allow us to assess which of the different training components was responsible of the observed effects, it seems to us that the applicative goal of designing an effective intervention was attained.

### Near transfer effects

Turning to the findings concerning “transfer effects,” our experimental cognitive training program, unlike most other types of working memory treatments, consisted of highly varied activities never involving the same type of verbal processing (i.e., judging semantic plausibility) or to-be-memorized-units (i.e., the last word of each sentence) required by the listening span test. Thus, Davide's improved performance with the listening span test was a reflection of “near” transfer to an untrained task.

We should also emphasize some absence of near transfer effects emerging from the follow-up assessment, the first consisting of a lack of improvements with backward digit span and the second of a very low increase of episodic memory. Lack of training effects on performance with backward digit span may be explained by taking into account that Davide did not practice at all the specific type of processing (i.e., repeating items in the reverse order) involved in backward digit span in our cognitive training program. As such, lack of practice had a negative impact on his post-test performance. This suggests that working memory ability, though improved, was not sufficient to prevent the child's difficulty with a type of verbal processing that he had not been practicing.

Turning to episodic memory, the very low improvement of performance in a narrative memory task may suggest that the episodic buffer—although the target of some complex working memory activities in our program—was not affected by training. According to Baddeley (2000), this particular working memory component depends on executive processing, but is primarily concerned with the storage of information rather than with attentional control. It is not clear yet to what extent binding together information from different sources into chunks or episodes depends on activation of concepts and schemas from long-term memory or from a fluent coordinated working of executive processing, as well as visual and verbal short-term storage. The results of a study by Hambrick and Engle (2002)—which showed that knowledge of the topic influenced performance on retention of narrative passages much more than working memory—should be considered in interpreting Davide's performance on narrative memory. Such performance might have been more related to lack of expert knowledge on the stories' topics than to verbal working memory. Alternatively, the low modifiability of Davide's narrative memory may suggest that binding together information from different sources is a structural impairment for some individuals with intellectual disability, and is therefore very resistant to intervention. A deficit in binding together information may not impair performance when the instructions enforce both attentional control and explicit memory encoding, which occurs in the listening span test. Such a deficit is likely to generate an extremely poor episodic memory when the task does not have these characteristics, as when the instructions ask participants to listen to a story for later recall. This point deserves further exploration in future research as the binding of information into chunks or episodes is of the greatest importance in learning, and a deficit in this area may shed a less optimistic light on the transfer effects that can be generated by a more effective working memory in individuals with intellectual disability.

### Far transfer effects

Our study also explored whether “far transfer” effects of our combined treatment can be generated on cognitive flexibility that was assessed with a task engaging both concept formation and shifting (i.e., Animal Sorting from the Nepsy II). We found that Davide's performance in this task increased of one standard deviation in the follow-up testing. Thus, there was a slight far transfer effect to more flexible processes of concept formation and shifting. Moreover, as the scores in the Animal Sorting test correlate most highly with Matrix Reasoning (0.49, as reported in Korkman et al., 2007, p. 89), an increased capability of addressing problem-solving tasks may complement the increase in cognitive flexibility.

In our opinion, Davide's improvement in concept formation and shifting should be interpreted as related not only to the enhanced cognitive mechanisms but also to the more benevolent beliefs about himself that started to emerge in the conversations occurring in the initial stage of our cognitive training sessions (Fatigante et al., 2015). It is well known that holding either a fixed or an acquirable view of intelligence deeply affects a student's performance on learning tasks (Mangels et al., 2006). Individuals with fixed view of intelligence are more likely to avoid learning situations where they anticipate a high risk of errors. In tasks such as Animal Sorting, in which participants are asked to think of different possible ways for grouping images that are quite dense in visual details, individuals who do not trust their own thinking and problem-solving abilities are likely to have a poor performance.

Although Davide still tended to present himself as a non-intelligent person after almost 2 years of intervention, he could smile while saying “I'm not intelligent,” and somehow waited for the therapist's questioning of such “old” belief (see the dialogue reported in Table 6). Contrary to this, in the initial dialogues, he positioned himself as hostile, helpless, or discouraged toward his reasoning abilities. Davide has been constantly reminded of the idea that intelligence is a kind of power that is within every human being and that manifests itself thanks to the help of a wide range of more-specific abilities, such as attention, language, and memory.

**Table 6:** **A dialogue between Davide and the therapist (MO)**.

The excerpt is from a conversation focused on choosing a new professional high school after a first year in which Davide attended a professional school that he did not like. The doubt has to do with whether to move to the first-year class or second-year class of the new school.
• Therapist: Beh, se ricominci dal primo anno avresti due anni di più dei tuoi compagni (Davide è andato a scuola un anno più tardi).
*Well…if you start again from the first class you will find mates that are 2 years younger* (Davide started primary school 1 year later than his peers did.)
• Davide: Tanto non-importa, tanto anche se c'ho due anni in più, gli altri sono sempre più intelligenti.
*Well…It does not matter, even if I'm 2 years older…the others are always more intelligent*.
• Therapist: Che cosa? Che hai detto? (scherzando, marcando esageratamente le espressioni del viso)
*What? What did you say? (joking and with marked visual expressions)*
• Davide: Che anche se c'ho due anni in più, gli altri sono sempre più intelligenti.
*That even if I am 2 years older than my mates they are always more intelligent*.
• Therapist: Tu pensi questo? Pensi questo?
*Are you really thinking this? Do you think this?*
• Davide: (sorride)
*(he smiles)*
• Therapist: Sono più intelligenti in tutto?
*Are they more intelligent in everything?*
• Davide: Sì. (sorride)
*Yes. (smiling)*
• Therapist: (Abbassa la testa e fa un lungo sospiro.) Ma io vorrei sapere perché…noi lavoriamo tanto e tu però pensi sempre queste cose negative, Davide.
*(She lowers the head and sighs.) Davide, I would like to know why…we are working so much and you are still thinking such negative things of yourself*.
• Davide: Non lo so. (sorride)
*I do not know. (smiling)*
• Therapist: Ma tu spiegami una cosa, non c'è una cosa in cui ti senti intelligente?
*But tell me, is there a thing in which you feel you are intelligent?*
• Davide: Quando faccio le cose da solo mi sento intelligente.
*When I do things by myself I feel I am intelligent*.
• Therapist: Ah…e come mai allora?
*Ah, and why then?*
• Davide: Quando non so le cose non mi sento.
*When I do not know things I do not feel so*.
• Therapist: Ah, quando non sai le cose pensi “non sono intelligente.” Invece non è che pensi “non so le cose perché le devo ancora imparare.” Non è che pensi che puoi imparare, non lo pensi mai questo, che puoi imparare?
*Ah, when you do not know things you think “I'm not intelligent.” But you do not think “I do not know things because I still have to learn them.” You do not think you can learn, do you? Do you ever think that you can learn?*
• Davide: Non l'ho mai pensato. (sorride)
*I never thought this. (smiling)*

## Concluding remarks

This study explored the effects of training in which attention, inhibition, switching, and the ability to engage in elaborate processing and memory encoding with verbal tasks were stimulated. The main finding was that Davide, a 14-year-old boy with a mild intellectual disability, shifted from being incapable of addressing a verbal dual task such as the listening span test to having a performance close to the normal limits of a 13-year-old boy in the follow-up assessment with this test, when he was 15 years old. It should be emphasized that Davide's initial verbal short-term memory was low but within normal limits, whereas his ability to carry out dual working memory tasks was completely absent. Thus, our study shows on one hand a very encouraging finding, as deficits in verbal WM are often deeper than visuo-spatial deficits in children with intellectual disabilities (Henry and MacLean, 2002; Van der Molen et al., 2009; Soderqvist et al., 2012). On the other hand, such a good response to intervention on verbal working memory is likely to be also related to a specific individual characteristic: that of a verbal short-term memory that was not severely impaired.

The findings of our study induce an optimistic view of the cognitive modifiability of verbal working memory in children with intellectual disability, but more evidence is needed on the individual characteristics that may predict a good response to intervention. Further investigation is also required to analyze the far transfer effects of improved verbal working memory, clarifying whether or not a range of cognitive processes—from concept formation to discourse comprehension and verbal reasoning—can be positively affected.

### Conflict of interest statement

The authors declare that the research was conducted in the absence of any commercial or financial relationships that could be construed as a potential conflict of interest.
